# Letter to the Editor in Response to: Eagle SR, Henry RJ. Applying Dynamical Systems Theory to Improve Personalized Medicine Following Mild Traumatic Brain Injury. *Neurotrauma Rep* 2024 Jul 16;5(1):671–679; Doi: 10.1089/Neur.2024.0040

**DOI:** 10.1177/2689288X251377004

**Published:** 2025-10-23

**Authors:** Tim W. McGlennon

**Affiliations:** Biostatistician, Psychometric Research Analyst, Division of Biostatistics, McGlennon MotiMetrics, LLC (M3), Maiden Rock, Wisconsin, USA.

*Dear Editor:*

We read with great interest the article by Eagle and Henry^1^ in which the authors discuss applying dynamical systems theory to improve personalized medicine following traumatic brain injury (TBI). Using comorbid obesity/TBI as an illustrative example of a dynamical system, the authors explore preclinical and clinical evidence of multiple system interactions linking the two disease states. Foundational clinical evidence was derived from our group’s integrative review published (2020–2021) in *Obesity Surgery*.^2^

The review considered shared pathophysiological processes and symptomatology characterizing the obesity/TBI dynamical system and hypothesized that metabolic-bariatric surgery (MBS) may provide a unique multifactorial treatment modality for millions of obese individuals with chronic TBI.^1,2^ Subsequently, two noteworthy studies have found evidence supportive of the hypothesis that MBS may mitigate TBI. Henry et al. (2023) identified bidirectional, interactive effects between adipose tissue and the brain in obesity/TBI.^3^ These effects included additive amplifications of inflammatory pathways (e.g., NLRP3) leading to exacerbated neurocognitive dysfunction. Eagle et al. (2024) found evidence that obesity interacted with higher acute concentrations of NLRP3 inflammasome proteins and worsened long-term TBI outcomes.^4^

Due to the bidirectional nature of obesity/TBI and that adipose tissue has provided stronger evidence than the brain for inflammatory amplification and resultant cognitive dysfunction in chronic TBI, Henry et al. hypothesized that targeting visceral adipose tissue in obesity/TBI might represent a viable therapy for these patients.^3^ In their recent article,^1^ Eagle and Henry suggest that the majority of TBI research has been reductionistic, focused on treating the injury itself, and that treating complex organ systems below the cervical spine may improve TBI outcomes.^1^ Accordingly, MBS appears uniquely qualified to positively influence the course of chronic TBI in this cohort. MBS is the most effective treatment for obesity, and it also induces favorable physiological changes across multiple organ systems. MBS has been shown to reverse inflammation in visceral adipose tissue by suppressing NLRP3 inflammasome activation,^5^ the very mechanism thought to be causal in exacerbating TBI pathology, and pinpointed as a novel therapeutic target in obesity/TBI.^1,3,4^

There are no FDA-approved pharmacotherapies for the treatment of chronic TBI.^2^ Although it is counterintuitive that an operation on the stomach could benefit the brain, research demonstrates that MBS is protective of cognition and activates the GLP1-SGLT1 signaling pathway in the hippocampus, attenuates microgliosis and neuroinflammation, confers neuroprotection by elevating adiponectin levels, inhibits beta-amyloid and tau accumulation, and induces white and gray matter density recovery in patients with obesity.^2^ Most interestingly, MBS also ameliorates chronic TBI symptomatology (i.e., cognitive impairment, migraine headache, sleep disorders, and mood disorders).^2^

An innovative concept tested by a surgical procedure has the potential to elicit discoveries translatable to non-surgical therapy. MBS perhaps offers the most powerful intervention currently available to: (1) effectively treat obesity-related diseases and TBI symptomatology in this cohort and (2) elucidate metabolic-inflammatory causal interactions underlying the obesity/TBI dynamical system. These findings may lead to pharmacotherapies that modify the brain’s long-term response to TBI in the general population.
